# Polyarticular Septic Arthritis Due to Non-Typeable *Haemophilus influenzae* With Concomitant New-Onset Acute Gouty Arthritis

**DOI:** 10.1177/2324709619864990

**Published:** 2019-07-25

**Authors:** Brian Sanders, Mohammed Abdulfatah, Mossab Aljuaid, Ibrahim Tawhari

**Affiliations:** 1 University of Utah Health, Salt Lake City, UT, USA

**Keywords:** arthritis, *Haemophilus*, gout

## Abstract

*Haemophilus influenzae* is serologically classified into two main categories based on the presence or absence of the polysaccharide capsule. Strains that possess polysaccharide capsules are identified as typeable *Haemophilus influenzae*, whereas strains that do not have capsules are identified as non-typeable *Haemophilus influenza*. Only on very rare occasions, *Haemophilus influenzae* affects adult joints, and almost 95% of cases have been identified as type b serotypes. Coexistence of gouty and septic arthritis is rare but has been reported. We herein report a case of polyarticular septic arthritis caused by non-typeable *Haemophilus influenzae* in an adult with concomitant new-onset gouty arthritis. The case was successfully treated with surgical debridement and a 4-week course of ceftriaxone.

## Introduction

*Haemophilus influenzae* commonly colonizes the human respiratory tract. It is serologically classified into 2 main categories based on the presence or absence of the polysaccharide capsule. Typeable strains are strains that possess polysaccharide capsules, whereas non-typeable strains do not have capsules.^1^ Only on very rare occasions, *H influenzae* affects adult joints, and almost 95% of cases have been identified to have been caused by type b serotypes.^2^ Coexistence of gouty and septic arthritis is rare but has been reported. In this article, we report a case of polyarticular septic arthritis caused by non-typeable *H influenzae* in an adult with new-onset gouty arthritis.

## Case Presentation

A 56-year-old man presented to the emergency department with a 5-day history of painful and swollen wrists, ankles, and right knee with associated fever and limitation of movement. He was seen at the primary care clinic 2 days prior to presentation at the emergency department and was diagnosed with gouty arthritis based on elevated serum uric acid level. He was also started on prednisone due to the diagnosis of acute kidney injury. The pain and swelling continued to worsen with spreading erythema around the ankles and right knee; he therefore decided to seek further evaluation at our emergency department. His past medical history was notable for essential hypertension for which he used lisinopril.

His body temperature was 38.2°C, heart rate was 108 beats per minute, respiratory rate was 12 breaths per minute, and blood pressure was 135/66 mm Hg. Physical examination revealed erythema and swelling with fluctuance over both wrists, ankles, and right knee with localized tenderness, warmth, and limited range of motion.

A complete blood count revealed a leukocyte count of 23.53 × 10^9^/L with 86% neutrophils and 26% band cells, a hemoglobin concentration of 14.8 g/dL, and a platelet count of 105 × 10^3^/µL. Blood chemistry indicated a creatinine level of 1.58 mg/dL, blood urea nitrogen level of 71 mg/dL, anion gap of 17 mmol/L, bicarbonate concentration of 18 mmol/L, and lactate concentration of 1.7 mmol/L. His C-reactive protein level was 48 mg/dL. Synovial fluid analysis revealed a leukocyte count of 118 000/µL, 98% polymorphonuclear cells, red blood cell count of 100/µL, and a positive test for monosodium urate (MSU) crystals. Gram staining revealed Gram-negative coccobacilli. Synovial fluid culture grew *H influenzae*, which was identified as non-typeable by polymerase chain reaction. Transcription-mediated amplification yielded negative results for chlamydia and gonorrhea. Two sets of blood cultures yielded no significant findings.

The patient was empirically started on vancomycin and piperacillin/tazobactam. He received a loading dose of intravenous (IV) vancomycin 20 mg/kg, which was discontinued shortly when synovial fluid gram staining revealed Gram-negative coccobacilli. Piperacillin/tazobactam was started at 2.25 g intravenously every 6 hours, adjusted for his renal function. The patient immediately underwent irrigation and debridement of the right knee, both ankles, and both wrists. The antibiotic spectrum was subsequently narrowed down to ceftriaxone 2 g intravenously every 24 hours as synovial culture revealed susceptibility to ceftriaxone. Due to impaired renal functions on presentation, the patient received 3 L of IV normal saline and lisinopril was held for 2 days until renal function had recovered. Renal function normalized next day, and the patient received a 3-day course of indomethacin 50 mg orally every 8 hours for the acute gouty arthritis.

He was discharged on ceftriaxone and completed a total of 4 weeks of treatment. At follow-up 4 weeks later, the joint swelling, pain, and erythema had resolved and the patient was able to move his joints without any limitations.

## Discussion

*Haemophilus influenzae* is a small, pleomorphic, oxidase positive, facultative anaerobic, and non-motile Gram-negative coccobacillus. It commonly colonizes the human respiratory tract and is transmitted through droplets and contact with respiratory secretions. The presence or absence of a polysaccharide capsule is an important characteristic of this organism. The species that possess polysaccharide capsules are referred to as typeable *H influenzae* and serologically classified into 6 serotypes (*a* through *f*). On the other hand, the species that do not possess a polysaccharide capsule are considered non-typeable *H influenzae* (NTHi).^2^

In general, only on very rare occasions, *H influenzae* causes invasive septic arthritis in adults, and majority of serotyped cases have been identified as type b.^2^ Since the introduction of the *H influenzae* type b vaccine, most cases of invasive *H influenzae* infection have been caused by non-typeable b strains, with serotype f and NTHi being the most commonly reported.^3^ It has been suggested that the presence of underlying medical conditions that impair the reticuloendothelial system, a network of phagocytic cells found throughout the body, particularly in the blood, general connective tissue, spleen, liver, lungs, bone marrow, and lymph nodes, may predispose patients to invasive diseases caused by NTHi infection. We searched PubMed and Google Scholar to identify previously published cases in the English language using the keywords “*Haemophilus*,” “non-typeable,” “septic,” “arthritis,” and “joint.” We found only 6 published cases of septic arthritis caused by NTHi strains in adults. The underlying predisposing medical conditions affecting the immune system in 5 of 6 cases included multiple myeloma, alcoholic liver disease, common variable immunodeficiency, rheumatoid arthritis, steroid therapy, and laryngeal cancer, as summarized in Table 1. However, one of the cases had no predisposing factors.^4-9^ Our patient did not have any predisposing factors affecting the immune system, but had concomitant new-onset acute gouty arthritis.

**Table 1.** Summary of Reported Adult Cases of Non-Typeable *Haemophilus influenzae* Septic Arthritis With Antibiotic Susceptibility.

Reference	Age, Years	Sex	Affected Joint(s)	Predisposing Factors	Susceptibility	Treatment	Outcomes
Our case	56	Male	Wrists, ankles, and right knee	Gout	Ceftriaxone, fluoroquinolone, ampicillin, and cefuroxime	Ceftriaxone + surgical debridement	Resolved
Kim et al^4^	18	Male	Polyarticular	None	Not available	Ampicillin + arthrocentesis + surgical debridement	Resolved
Saba et al^5^	46	Female	Both knees, right ankle, right elbow, and left wrist	Multiple myeloma	Ampicillin, penicillin, and chloramphenicol	Ampicillin + arthrocentesis	Resolved
Lester et al^6^	40	Male	Both knees, both ankles, left elbow, and left wrist	Alcohol use	Ampicillin, cefuroxime, and netilmicin	Cefuroxime + arthrocentesis	Resolved
Hawkins et al^7^	35	Male	Left knee	Common variable hypogammaglobulinemia	Aztreonam, gentamycin, and mezlocillin	Aztreonam + arthrocentesis	Resolved
Melhus and Svernell^8^	46	Female	Right shoulder, right elbow	Rheumatoid arthritis, right elbow steroid therapy	Ceftriaxone, ciprofloxacin, and netilmicin	Ciprofloxacin + arthrocentesis	Resolved
Turner et al^10^	57	Male	Right knee, left wrist	Laryngeal cancer, gout, smoking, and alcohol use	Ampicillin, augmentin, azithromycin, cefuroxime, levofloxacin, and Bactrim	Ampicillin + surgical debridement	Resolved

Coexistence of gouty and septic arthritis has been reported. Several mechanisms for their coexistence have been reported. Joint infection results in an increased local production of lactic acid due to the influx of neutrophils with a fall in pH that subsequently decreases the solubility of MSU and further results in crystal precipitations. Other than pH, certain plasma proteins and polysaccharides altered by sepsis could also contribute to the precipitation of MSU crystals.^10^ This indicates the importance of thorough evaluation of aspirated synovial fluid.

The ideal duration of antimicrobial therapy for septic arthritis remains uncertain. The recommended total duration of postsurgical antibiotic therapy ranges between 2 and 6 weeks, with most clinicians recommending 4 weeks. This recommendation is based on expert opinions, and there are no available randomized trials. Our patient received 4 weeks of IV antibiotic therapy based on Infectious Disease expert opinion in our hospital. A recent prospective unblended randomized noninferiority study concluded that 2 weeks of targeted antibiotic therapy is not inferior to 4 weeks in terms of the cure rate following surgical lavage for septic arthritis.^11^
